# Real-life impacts of olipudase alfa: The experience of patients and families taking an enzyme replacement therapy for acid sphingomyelinase deficiency

**DOI:** 10.1186/s13023-024-03020-4

**Published:** 2024-02-01

**Authors:** Eva M. Raebel, Samantha Wiseman, Conan Donnelly, Toni Mathieson, Jackson Pountney, Joslyn Crowe, Justin Hopkin

**Affiliations:** 1 Rare Disease Research Partners, MPS House, Repton Place, White Lion Road, Amersham, HP7 9LP UK; 2 International Niemann-Pick Disease Registry, Suite 2 Vermont House, Washington, Tyne and Wear, NE37 2SQ UK; 3 Niemann-Pick UK, Suite 2 Vermont House, Washington, Tyne and Wear, NE37 2SQ UK; 4 Institute of Health and Neurodevelopment, College of Health and Life Sciences, Aston University, Birmingham, B4 7ET UK; 5 NNPDF, PO Box 49, Fort Atkinson, WI 53538 USA; 6 Rochester, USA

**Keywords:** Acid sphingomyelinase deficiency, Olipudase alfa, Enzyme replacement therapy, Niemann Pick diseases, Type A, Type A/B, Type B, Visceral symptoms, Neurologic symptoms

## Abstract

**Background:**

Acid Sphingomyelinase Deficiency (ASMD) is an ultra-rare autosomal recessive lysosomal storage disorder characterized by intracellular lipid accumulation resulting from reduced function of acid sphingomyelinase. Olipudase alfa, an enzyme replacement therapy, was recently approved in several countries for the treatment of the non-neurologic manifestations of ASMD. Studies demonstrate improvement in organomegaly, pulmonary function and lipid profiles with olipudase alfa, yet little is known about its impact on quality of life (QoL) for patients and caregivers. The purpose of this study is to better understand the real-life impact of ASMD on patients and caregivers and assess how olipudase alfa impacts QoL for pediatric patients and their caregivers.

**Methods:**

Caregivers of pediatric patients (≤ 18 years of age) with a confirmed diagnosis of ASMD that received olipudase alfa for at least 12 months were recruited in early 2022 through national patient organizations to participate in a global online questionnaire followed by semi-structured interviews. Ten caregivers of patients with ASMD who utilized olipudase alfa as an experimental therapy for pediatric patients participated in the study. Quantitative analysis of the results was undertaken, and qualitative data was analyzed using an inductive thematic approach.

**Results:**

Ten eligible participants completed questionnaires, and 8 of the 10 went on to participate in structured interviews. Symptom burden of ASMD and impact on symptomatology and quality of life after olipudase alfa use are reported here. Five themes emerged from analysis: (1) ASMD is a systemic disease with a wide array of manifestations that significantly impact QoL; (2) Olipudase alfa was associated with improvements in all non-neurologic manifestations of ASMD; (3) Participants perceived the risk associated with olipudase alfa to be low and the benefits to greatly outweigh any risk or burden; (4) Participants reported an unmet need to treat the neurologic manifestations of the disease despite the benefits of olipudase alfa in the management of non-neurological symptoms; (5) Participants felt all patients with ASMD need access to olipudase alfa based on the life-changing experience they perceived.

**Conclusions:**

These findings highlight the sustained positive impact olipudase alfa had in many domains that are deemed important to patients and families living with ASMD and outline the extensive unmet need for patients and families living with ASMD.

## Introduction

Acid sphingomyelinase deficiency (ASMD, Niemann-Pick) is a rare autosomal recessive lysosomal storage disorder (LSD) caused by mutations in the *SMPD1* gene, which codes for ASM [1]. Deficient ASM activity results in the accumulation of sphingomyelin and other lipids in cells and tissues [2]. ASMD is commonly classified as acute neurovisceral (also known as Niemann-Pick disease type A) or chronic visceral (also known as Niemann-Pick type B) or chronic neurovisceral (also known as Niemann-Pick Type A/B) [3]. ASMD is heterogenous in phenotypic presentation and disease severity, with type B associated with minimal to no central nervous system involvement [4], and type A/B presenting with some neurological involvement but not as severe as type A [5]. Type A is characterized by severe progressive neurodegeneration in the first years of life followed by early death. Clinical features related to the early neurological dysfunction include irritability, sleep disturbance, failure to thrive, hypotonia, severe gastrointestinal and progressive respiratory symptoms [6, 7]. Clinical features of type B were reported in an international cross-sectional study of 59 patients and included splenomegaly (78%) and hepatomegaly (73%) at presentation while pre-existing complaints included bleeding (49%), shortness of breath (42%), pulmonary infections (42%), joint and/or limb pain (39%), bruising (27%), headaches (24%), diarrhea (20%) and bone fractures (19%). In addition, most patients with type B had below-average height and weight which was most pronounced in adolescence but appeared to approach normal values in early adulthood [7].

There is a paucity of literature documenting the birth prevalence of ASMD with wide variation in estimates. A 2015 study [8] of lysosomal storage disorders conducted across six different countries during 1999–2013 reported a live birth prevalence of 0.25–0.6/100,000. In contrast, a study in Chile involving screening of almost 1700 healthy individuals for a common *SMPD1* pathogenic variant, p.Ala359Asp, found a heterozygote frequency of 1:105.7, predicting a much higher disease incidence of 1:44,960 [9]. Variants causing the severe neurodegenerative form type A disease are more common among the Ashkenazi Jewish population contrasting with the pan-ethnic distribution of later-onset and mild forms of acid sphingomyelinase deficiency (type B) [9, 10].

Olipudase alfa is an enzyme replacement therapy (ERT) that has recently been approved in several countries to treat the non-neurologic manifestations of ASMD as it does not cross the blood brain barrier [11]. In the clinical trials, both pediatric and adult ASMD patients had clinically significant reductions in spleen and liver volume, improvements in lung diffusion capacity (DLCO), and improved lipid profiles associated with olipudase alfa [11, 12]. In addition, significant increases in height based on the age of pediatric patients was reported [11].

While the clinical outcomes associated with olipudase alfa are promising, there is a need to examine the impact of therapy on the patient-reported experience of disease burden and QoL to better understand the value of olipudase alfa on the patient, caregiver and family.

No studies to date have explored the patient or caregiver perspective of patients who have experience with the experimental therapy olipudase alfa. The National Niemann-Pick Disease Foundation (NNPDF), Niemann-Pick UK (NPUK) and the International Niemann-Pick Disease Registry (INPDR) aimed to understand the impact of olipudase alfa therapy on families affected by ASMD from a caregiver perspective. This study aimed to document: (1) the burden of ASMD, (2) the patient and caregiver perspective of ASMD, (3) what is perceived as meaningful in a therapy, (4) the risk tolerance to a potential therapy and (5) the unmet therapeutic needs for ASMD patients.

## Method

This study was a retrospective case series study that obtained both qualitative and quantitative data through online surveys and semi-structured interviews co-designed by researchers, clinicians, patient advocates and patient families, taking into consideration known natural history of the disease [7, 9]. Rare Disease Research Partners (RDRP) conducted the data collection on behalf of NNPDF, NPUK and INPDR.

### Recruitment

NNPDF and NPUK shared communication with their own members as well as other national member organizations about the study via email. In addition, NNPDF and NPUK emailed eligible participants in each country based on mailing lists and previous communication with each national patient organization.

There were 20 participants in the ASCEND-Peds trial, a global trial with six sites and patients on three continents [11]. The recruitment goal for our study was to include as many participants from the trial as possible while also recruiting any pediatric patients who had significant experience with olipudase alfa through a Managed Access Program according to the eligibility criteria (Table 1). The final sample size of ten is sufficient for an in-depth and detailed exploration of participant experience [10].

**Table 1:** Participant inclusion criteria

Parent/caregiver^a^ (or study participant if ≥ 18 years of age at the time of enrollment in our study)	&
Fluent in English (including non-native English speakers)	&
Able to give informed consent	&
With a confirmed diagnosis of ASMD	&
Used the experimental drug olipudase alfa for more than 12 months	&
Commenced treatment with olipudase alfa under the age of 18 years	&
Consented to participate in the study	

^a^Hereafter referred to as caregiver

This research was conducted in accordance with the British Healthcare Business Intelligence Association’s Legal & Ethical Guidelines for Market Research.

Caregivers with more than one child with ASMD receiving olipudase alfa were asked to complete a separate survey for each child.

### Study design

The online survey was co-designed by NNPDF, NPUK & INPDR to explore demographics, first symptoms, symptoms before and after treatment, overall change in symptoms and activities since treatment, current treatment regimen for ASMD, and experience with olipudase alfa. Given the lack of validated tools to assess patient reported outcomes (PRO) for ASMD patients and families, the authors convened key opinion leaders, researchers, families, patients, and advocacy organizational leaders to develop additional survey questions. The survey included multiple choice, matrix and open text questions to provide both quantitative and qualitative data. The survey included questions that closely resembled the Splenomegaly Related Scale (SRS) used in the adult ASCEND trial to assess disease burden that may be related to organomegaly. The survey was hosted on the online Qualtrics^XM^ platform [13] and distributed via a link. A Participation Information Sheet and Informed Consent were displayed at the start of the online survey. Only those that provided consent were able to proceed with the survey. The survey was open from 19 January to 15 February 2022. No formal sample size calculation was performed for the study; the aim was to recruit as many patients as possible that met the eligibility criteria for the study.

The semi-structured interviews were available to respondents who had consented to be contacted by NNPDF about the interviews and had completed the online survey. Those who wished to participate in an interview were required to complete an additional consent form prior to the interview. The interview guide was developed to cover questions from the survey in more depth and to further understand the impacts of olipudase alfa on patients and their families. Interviews were conducted via Zoom© by one experienced, independent qualitative researcher contracted to RDRP. No monetary voucher or incentive was used to engage participants.

### Data analysis

Survey data involved a comparison of the proportion of patients reporting symptoms and experience before and after treatment. Interview transcripts were analyzed using an inductive thematic approach as described by Braun and Clarke in 2019 [14] using NVivo software. In brief, transcripts were reviewed and emerging themes were identified and coded independently by RDRP. The study methods aligns to guidelines for conducting and reporting qualitative research [15].

## Results

### Participant characteristics

Twenty-six respondents attempted the survey between January 19 and February 14, 2022. Sixteen passed the screening questions (4 did not continue with the survey, 2 respondents were adult patients and were excluded from this report) resulting in a total of 10 patients that were included in the quantitative analysis. Of these, 8 went on to participate in the qualitative component of the study. All respondents included in this study were caregivers of a patient with ASMD and included one caregiver who completed separate surveys for two children. Interviews were undertaken with the caregivers of eight patients, with one caregiver completing separate interviews for two children. Responses from this caregiver were different for each of the two children and have been analyzed separately. Where data referred to caregivers only, analysis has been performed on nine caregivers and this has been indicated.

Survey respondents were born and resided in four different countries: Belgium, Brazil, USA, and the UK. The ten patients included in the survey were between 4 and 17 years of age at the time of survey, with 8/10 between 4 and 12 years old. Median age of diagnosis of ASMD was 1 year of age (mean 1.7 ± 1.4, range 0–5). Treatment with olipudase alfa was started at a median age of 3.5 years of age (mean 5.0 ± 3.8, range 1.5–14.0 years). All patients were still on olipudase alfa at the time of the survey and had been on therapy for a median of 5 years (mean 4.6 ± 1.5, range 1.3–6.1).

### Overarching themes

Five overarching themes were identified from the data. The quantitative and qualitative findings are presented under each theme below:

***Theme 1***: **Symptom management**

**Fig. 1:**
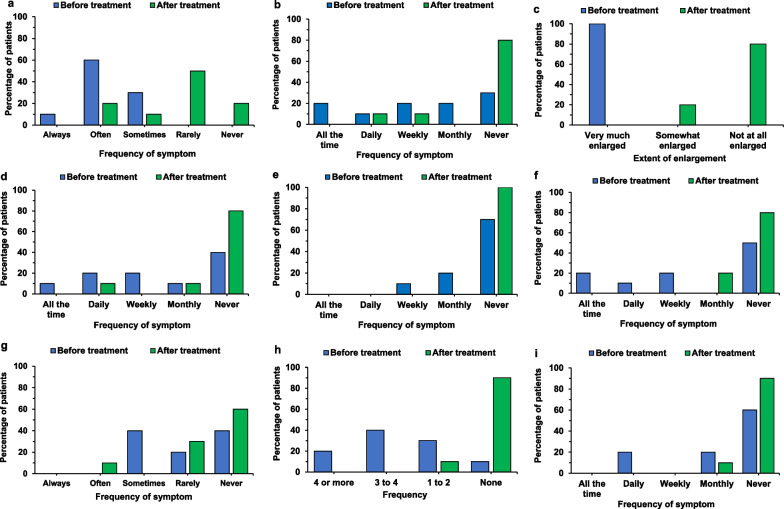
Survey reported frequency of ASMD symptoms before (n = 10) and after (n = 10) treatment with olipudase alfa. Symptom categories are tiredness and fatigue (**a**), abdominal pain (**b**), organ enlargement (**c**), vomiting and nausea (**d**), bone pain (**e**), bruising (**f**), chronic headaches (**g**), infections over a 6-month period (**h**), and shortness of breath (**i**)

ASMD is a systemic disease with a wide array of manifestations that has a meaningful impact on QoL. Survey results show olipudase alfa was associated with improvements in all non-neurologic manifestations observed by patients and families (see Fig. 1).

#### Fatigue

Fatigue was a common symptom reported in the survey with 9/10 participants noting fatigue either “often” or “sometimes” prior to initiating olipudase alfa treatment. Fatigue improved for all participants as illustrated in Fig. 1 and is supported by interview data from caregivers. One caregiver stated olipudase alfa treatment resulted in their child being more energetic, participating in more sporting activities and improved ability to cope with demands in educational settings:

*Before treatment:*

“…And as time passed by, we noticed that her energy level decreased, and when she got home from school, she asked us to go to bed.[…] And we also noticed that if she had to walk a certain distance, that it was difficult for her to catch up with the other children because she was always tired.”

*After treatment:*

“Also, the energy, she is really full of energy now. It’s amazing. She’s very active and she likes to do sports, […] Now she’s really an early bird, she’s awake very early, and it’s not a problem for her to handle these long schooldays anymore.”

*Interview, started treatment at 4 years of age, 3 years and 6 months on treatment*

#### Abdominal pain and organ enlargement

Abdominal pain was a common symptom of ASMD, noted to be present in 7/10 survey participants at baseline. Abdominal pain improved after treatment with olipudase alfa, with only 2/10 participants experiencing symptoms at the time of the study. There was also a marked difference in perception of organomegaly, with all participants noting the abdominal organs were “very much enlarged” prior to treatment. After olipudase alfa, 8/10 participants noted no enlargement and 2/10 noted somewhat enlarged organs. The difference before and after olipudase alfa treatment in participants’ experience of abdominal pain and organ enlargement was commented on by caregivers in interviews:

*Before treatment:*

“But his organs, his belly was becoming increasingly more distended before the ERT […]. He was hooked up to feeding pumps because the pressure that was being put on his stomach, he was only able to tolerate small volumes at a time. He was on continuous feed, so he had a feeding pump hooked up to him all the time, and oxygen hooked up to him. It’s just a lot of stuff for a little kid to deal with.”

*Interview, started treatment at 2 years of age*

*Before treatment:*

“…she complained about pain in the belly. So, that’s very difficult for us to exactly understand what it is that she feels, but I remember that every day she said, yes, my belly hurts, I have pain. Maybe it was the organs that were very dense. I don’t know. It was also difficult for her to exactly indicate where it was, but every day she told us that she was having pain in the belly.”

*After treatment:*

“Also, the pain in the belly is completely gone, I think it’s due to the organs that went to normal size again, and that is also supported by what the doctors tell us.”

*Interview, diagnosed at 2 years of age, started treatment at 4 years of age, 3 years and 6 months on treatment*

#### Nausea and vomiting

Half of the survey participants noted nausea and vomiting present prior to starting olipudase alfa, occurring at least weekly. After treatment, 8/10 participants experienced no nausea or vomiting.

*Before treatment:*

“Ninety percent of his calories came from a PediaSure or like a very thick liquid that he drank with a little bit of food on the side of that……It would be every third or fourth day he could have a vomiting episode, it just seemed like he would get backed up or too big a bite, something would cause the gag reflex in him.”

*After treatment:*

“About six months into treatment… We’d started watching him wolf down food and we were just ready for him to gag…All of a sudden, he’s just taking orange slices like nothing. And I think I just remember … holy cow, he’s just like downing food. And so, all of a sudden, he could eat no problem, everything and anything. We slowly phased out the fortified drink, it was probably decrease, decrease, decrease, it took a year.”

*Interview, started treatment at 6 years of age*

*Before treatment*

“He would eat a full meal and he would eat a lot. And then after he was finished eating, he would throw up, which it makes sense now, with everything being so enlarged. But yes, so he would eat. And that’s why I didn’t understand why he wasn’t gaining weight. Because he would eat a lot and then he would throw up, not at the end of every meal, but enough to make you wonder, why is this happening?”

*Interview, before treatment, started treatment at 1.5 years of age*

In surveys, all participants noted abdominal symptoms were “very much improved” compared to before starting olipudase alfa.

#### Growth delay

**Fig. 2:**
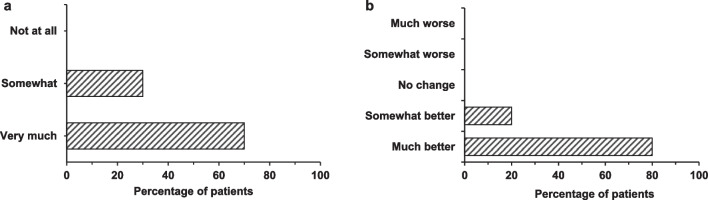
Survey reported extent of growth delay experienced by children before treatment with olipudase alfa (**a**) and changes in growth delay in response to treatment (**b**). Both cohorts were n = 10

As noted in Fig. 2, growth delay was common to all survey participants and every participant noted a positive impact on growth after olipudase alfa therapy.

The survey reported improvements in children’s heights and weights resulting from olipudase alfa treatment that were corroborated by caregivers’ interview responses. One parent in particular commented on how the treatment resulted in their child now being of healthy height and weight:

“Before beginning the drug, he was I think in the 15th percentile of his age for height and weight. He is now, I believe, in the 85th to… I think it’s the 85th percentile. But he has a head full of hair. He is over three feet tall. He is weighing about 40 lbs. He’s a little skinny but he’s tall.”

*Interview, started treatment at 1.5 years of age, 3 years and 10 months on treatment*

“He has grown a ton. He went from not being on the growth chart at all, to he’s now at the 50th percentile for weight and height. So, he grew a lot, put on a lot of weight, and he’s a healthy size now.[…] His body is just absorbing nutrients a lot better now.”

*Interview, started treatment at 2 years of age, 1 year and 3 months on treatment*

***Theme 2*** **:** **Emotional and mental health impacts**

The emotional and mental health impacts of ASMD on caregivers were extensive, and survey participants reported that olipudase alfa mitigated those impacts in a meaningful way. The majority of caregivers (9/10) responded that ASMD had an impact on their mental health, with stress, anxiety and depression being reported by the majority. Caregivers also reported impacts on their family and social life, on their relationships with partners, family and friends, and on their physical health.

#### Mental health impacts

**Fig. 3:**
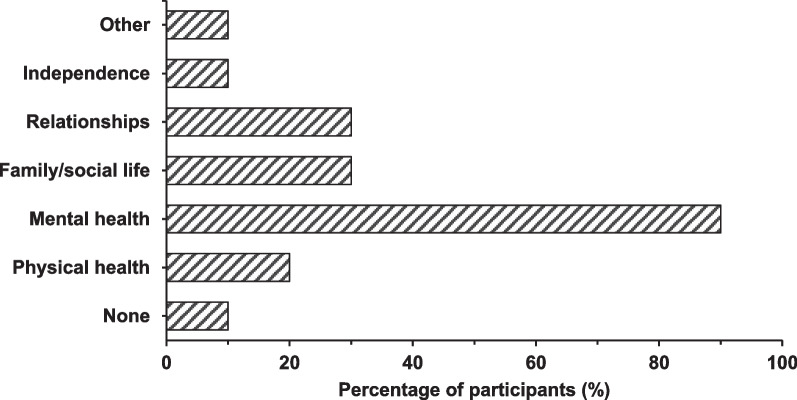
Survey reported impact of ASMD on caregivers’ wellbeing before olipudase alfa treatment (n = 10). Percentages cumulatively are higher than 100% as respondents were able to report more than one impact

In the surveys, caregivers reported feelings of anxiety, stress and depression at baseline (Fig. 3). Caregivers experienced concerns about their child’s safety and health, noting personal feelings of isolation, guilt and fatigue exacerbated by increased caregiver responsibilities and lack of sleep related to the child waking up frequently at night. In addition, sadness was an issue for caregivers who saw their child not being able to “live a normal life.”

“It is heart-breaking to watch your child go through everything that he’s been going through. Really, it puts a lot of stress on us as parents, but also me and my husband’s relationship, and it affects all aspects of our lives. Before his infusions and stuff, he required so much care.” *Interview, before treatment, started treatment at 2 years of age*

“I think you think of them every day and you think of them every night. You wake up thinking about it. That takes over your life, how am I going to normalize my child’s life? How is she going to be able to live normal and not be constantly sick and in the hospital?” *Interview, before treatment, started treatment at 7 years of age*

A recurring theme was extreme psychological stress regarding the uncertain disease course and progression as well as the constant concern for worsening morbidity and death if the disease was left untreated.

“Extreme anxiety, extreme depression on my end, a lot of frustration. My husband and I, you go from living this typical life essentially to being thrown with a potentially life-threatening diagnosis of your child.”

*Interview, before treatment, started treatment at 3 years of age*

Some caregivers felt guilty because they passed on the abnormal genes associated with their child’s disease. They questioned whether the decision to have children was the right one since their children had an inherited condition that would adversely affect their QoL.

“It was dark times. We questioned and felt a lot of guilt and questioned did we do the right thing looking to have kids? Should we have done more genetic testing? Were we selfish to think that we thought we didn’t have these mutated genes in our cells? There was a lot of stress for us as parents just knowing that we brought kids into this world who were going to have an uphill battle.”

*Interview, before treatment, started treatment at 2 years of age*

#### Emotional exhaustion

Emotional exhaustion was a consequence of constantly waiting for ASMD to progress while trying to manage everyday symptoms.

“We already thought that he was on borrowed time, but I think there’s emotional exhaustion that you don’t even notice, you’re just kind of waiting for the ball to drop sort of, like the whole time you’re just waiting for him to lose an ability. […] you’re always looking for the regression.”

*Interview, before treatment, started treatment at 6 years of age*

“I can’t even tell you how mentally exhausting it is to clean up vomit so many times a day. It just wears on you, not even physically, but emotionally it’s really hard to just watch your child constantly throwing up, and just looking like they feel awful.”

*Interview, before treatment, started treatment at 2 years of age*

In addition to the challenges of caring for a child with ASMD, some families were also trying to negotiate other demands such as sustaining employment and parental responsibilities to their other children. This was overwhelming for caregivers who felt their own sense of well-being decline as a result of these pressures.

“We counselled ourselves, looking back on it, I wish we did [attend support groups or counselling]. But we were grinding, we were trying to maintain our jobs and we were trying to raise our other kid. And we didn’t cut out time for ourselves. But luckily, we were able to get through that. And my wife and I grew stronger as a team in that sense. I’m fortunate of that because it very easily could have gone another direction. We were so caught up in the grind of life and trying to do everything for our kids that we did not focus on ourselves.”

*Interview, before treatment, started treatment at 2 years of age*

Many caregivers described the sense of isolation they felt with a child that was medically fragile. This was evident in the trepidation caregivers felt with the prospect of leaving their child in the care of other family members because of the increased risk of a bad outcome with caregivers unfamiliar with managing the intricacies of the disease.

“At that point we really couldn’t leave [Name] with anyone but one of us. Both of our parents live close by, and they’re always willing and wanting to help, but there’s only so much they can do and so much that they’re comfortable with doing, just because of his health needs. Especially back then, we would always be scared if he was going to throw up and we’re not there, and what if he choked on it?”

*Interview, before treatment, started treatment at 2 years of age*

“We didn’t trust anybody to babysit her, we didn’t want anybody handling her even. She felt like almost like a fragile sheet of glass after she was diagnosed.”

*Interview, before treatment, started treatment at 3 years of age*

**Fig. 4:**
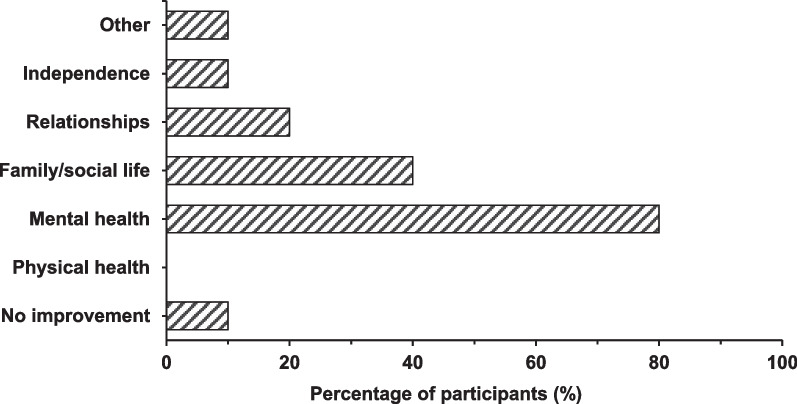
Impact of ASMD on caregivers’ wellbeing which were reported to have improved since olipudase alfa treatment (n = 10). Again, the sum of percentages is higher than 100% as survey participants were able to identify multiple aspects of wellbeing which improved

Most caregivers reported their stress and anxiety about their child’s health had lessened or even disappeared after treatment (Fig. 4). Interview responses from caregivers highlighted improvement in their own wellbeing resulting from their children’s treatment with olipudase alfa.

“We kind of breathed again. Before you just felt... so fearful to look forward to the future. Now we just, it’s hard to describe without getting emotional. Everything has just changed. It’s completely changed. We’re able to breathe again, we’re able to have hope again, we love sharing about it.[…] Our anxiety level has reduced tremendously; our depression has lifted.”

*Interview, started treatment at 3 years of age, 4 years and 11 months on treatment*

“And there’re also feeding pumps that are going off at night, and him waking up constantly, not sleeping well. Like you said, then we’re waking up constantly, and having to get up and clean up puke in the middle of the night, and then change bedsheets. It’s a lot. So, it’s definitely improved our sleep, too, our sleep habits, which is vital to your mental health.” *Interview, started treatment at 2 years of age, 1 year and 3 months on treatment*

“So, everything’s lifted in that sense that we’re thrilled knowing that our kids, during these prime years of the ages that they’re at, they’re able to do what any other kid is doing right now, and that means everything to us.”

*Interview, started treatment at 2 years of age, 6 years and 1 month on treatment*

**Fig. 5:**
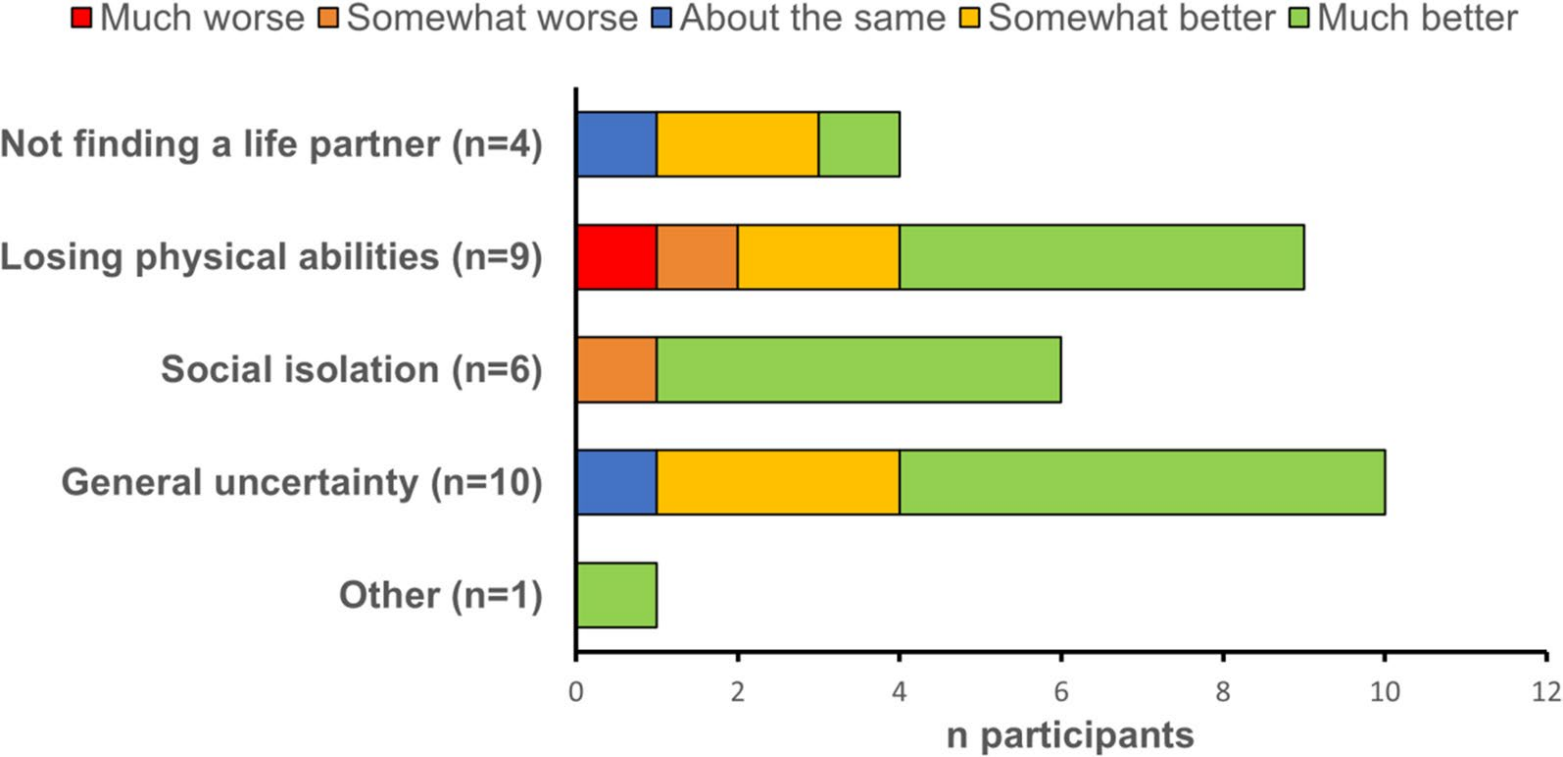
Number of survey participants reporting concerns about their child’s future before treatment and changes in these concerns following treatment

Caregivers also reported improvements in their concerns about the future of their child following treatment with olipudase alfa (Fig. 5).

Interviews with caregivers revealed that olipudase alfa treatment diminished concerns around the progression of their child’s disease and positively influenced their outlook regarding their child’s future.

“I've made a complete 180 now. I think she can be whatever she wants to be as long as she puts her mind to it. Everything’s opened up, and she wouldn't have had those opportunities if she hadn't been in the treatment.”

*Interview, 5 years and 9 months on treatment*

“We feel optimistic, we feel like we look forward to seeing the future. Where before we didn’t even think about the future, we didn’t think about what ifs, we didn’t think about what could be. And it’s amazing because [name] all she talks about is wanting to be a doctor because of all her experiences. So to see a child that was forced into a world of medical-ness as a necessary situation to now say that she knows that she wants to do the same thing to help little people like her.”

*Interview, 4 years and 11 months on treatment*

***Theme 3***: **Participant perception of risk**

Participants perceived little risk associated with olipudase alfa. In fact, they reported participation in the clinical trial to be the most significant burden or risk they encountered. The benefits perceived from olipudase alfa greatly outweighed any risk or burden.

“The hardest part of everything is the strict rules of the trial. The trial itself was the harder part than the drug itself. That is what caused the majority of the stress, getting the MRIs, getting the ultrasounds. And even something as little as them being done with their infusion and then having to wait an hour to get their vitals done, that hour time frame, that’s always been the hardest thing out there. Because the kids are done, they want to be up and go round. It’s just that extra hour after a four-and-a-half-hour infusion just adds to the point.”

*Interview, siblings, started treatment at 2 and 7 years old.*

When asked about concerns with olipudase alfa, most caregivers said in the survey that there were no disadvantages or adverse impacts on their child. Disadvantages that were mentioned in the survey included the treatment not crossing the blood–brain barrier, the child missing school on some days to receive the treatment and the demands and challenges of the clinical trial. In the interviews, all caregivers shared the perception that benefits of the treatment outweigh the risks, especially since most children had not suffered any kind of side-effect or reaction to the treatment. One parent/caregiver (first comment below) expressed uncertainty around the possibility of long-term side effects as part of the risk/benefit analysis but still felt the correct decision was made.

“I do still have concerns, maybe later in life, ten years down the road, will she have any side effects, I don’t know. I don’t think anyone knows. But at the same time, I would not take back the decision we made to have her in the trial because now she’s living the best life she can live. There’s absolutely no regrets there. And just thinking, the alternative there was her getting sicker and sicker. So, I definitely did a risk-benefit analysis before this all took place, and the benefits just seemed to far outreach the risk in her participating. Obviously, we were concerned, but very happy with the results, and look forward to her being a happy and successful adult.”

*Interview, 5 years and 9 months on treatment*

“The side effects for us were very mild and we had been through quite a bit with [Name] already, and his disease pretty progressed on the scale. So the slight things that happened at the beginning of the infusions, that didn’t faze quite a bit or at all.”

*Interview, 5 years and 8 months on treatment*

“We are absolutely thrilled with the treatment. The demands of the trial have presented numerous challenges; however, the benefits of the treatment greatly outweigh them.”

*Survey, 4 years and 1 month on treatment*

***Theme 4***: **Unmet therapeutic need**

Despite the overwhelming benefits of olipudase alfa, many participants cited an unmet need in managing the neurologic symptoms of ASMD. Identifying treatments for the neurologic manifestations of the disease remains a high priority for many patients and families.

**Fig. 6:**
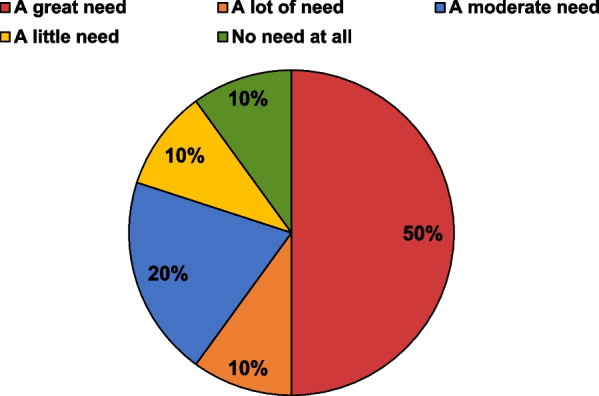
Caregivers’ survey response regarding the degree of need for a new treatment for children with ASMD (n = 10)

In the survey, caregivers were asked about their views regarding the level of need for a treatment other than olipudase alfa. Only one caregiver thought there was no need at all for a new treatment, but half of the respondents (5/10) perceived “a great need” and another 4/10 noted a degree of need (Fig. 6).

One caregiver reported that olipudase alfa would be sufficient to improve their child’s symptoms and that no additional treatment would be required.

“It may sound a bit selfish, and that’s not how I mean it, but in our case, in our case we don’t really see that need, because we see how she’s doing. And also, the medical specialists told us she is really doing very well. It’s unbelievable, because often in these types of diseases it’s very difficult to tackle all the symptoms, but in this case, it looks like it’s possible. So, they told us, we believe that as long as [Name] gets this treatment, she will do fine. From that point of view, we don’t really need other medication. I would say this treatment is perfect.” *Interview, 3 years and 6 months on treatment*

ASMD is a spectrum disorder with the variability in neurologic symptoms accounting for that spectrum. In this study just under half of survey participants perceived some sort of baseline neurologic abnormality they attributed to ASMD. This included hypotonia (40%), ataxia (30%), neuropathy (10%) and seizures (10%). Many participants suggested a need for a treatment that addresses the neurologic symptoms of ASMD.

Interviews with caregivers further highlighted the need for a treatment that addresses the neurologic sequalae of ASMD. Although participants acknowledged the positive impacts of olipudase alfa, the fact that it is not able to address neurological aspects of the disease was anxiety-provoking for some caregivers who expressed ongoing concern about the potential decline of their child’s condition.

“There’s just nothing treating the neurological portion of this disease that… It’s a fear but it’s a fear that we get to experience because he’s still doing great with us, so it’s just something that we are aware of because this treatment doesn’t help with the neurological things and we’re looking into other things. It’s just an unknown, we don’t know how long things will last with him or what it will look like in the future for him, but I would say more of an unknown. I mean the fear I mean is… We just don’t know what it will look like.”

*Interview, 5 years and 8 months on treatment*

“This drug has saved my son’s life, no doubt about that. We are so grateful and always will be, but it doesn’t cross the blood brain barrier to aid in neurological involvement.”

*Survey, 3 years and 10 months on treatment*

“I feel like this treatment is working beautifully at reversing damage done to their bodies and basically making their bodies normal again, but now the second piece to the puzzle is finding treatment to slow down or stop the neurological disease progression, or even reverse it. And it’s so important.”

*Interview, 1 year and 3 months on treatment*

***Theme 5***: **Access to olipudase alfa for the broader community**

**Fig. 7:**
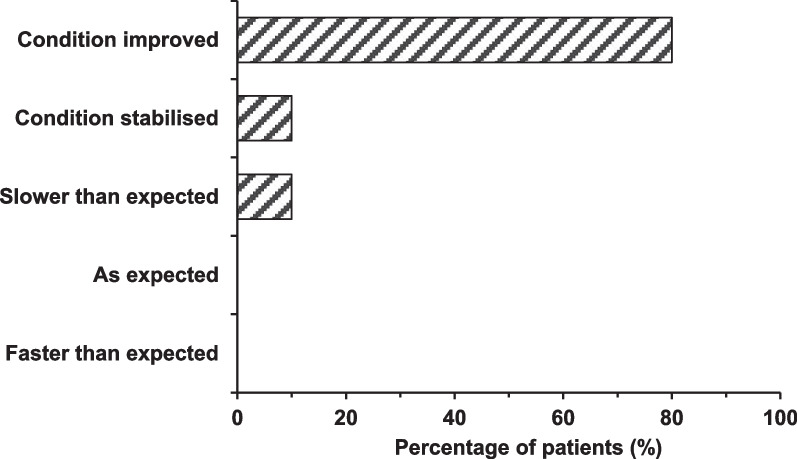
Caregivers’ survey response on the rate of decline or improvement or improvement in their child’s condition in response to treatment with olipudase alfa (n = 10)

Survey participants observed life-changing effects with olipudase alfa with 8/10 reporting that their child’s condition had improved because of treatment, whereas none of the respondents indicated that their child’s condition was progressing as would be expected without a treatment (Fig. 7).

Caregivers who were interviewed emphasized the significant improvement in their child’s condition while taking olipudase alfa. Particularly prevalent in interview responses was the establishment of ‘normalcy’ for their children. Their disease was not overtly recognizable compared to peers in the community to the extent that they could engage in play and other activities with peers.

“The best way to describe it is that she’s just like a typical child. Like when we share now that [name] has an underlying metabolic genetic disease people are totally shocked and surprised, I have to fill that information out when she attends like trampoline parks or attends school and teachers and people are so confused because they look at her and they’re like wait a minute what?”

*Interview, 4 years and 11 months on treatment*

“We have seen a drastic improvement in our child's physical, social, and mental health. He is able to engage and participate in age-appropriate play with his peers. He feels better about himself and his condition knowing that it can be treated.”

*Survey, 4 years and 1 month on treatment*

However, this establishment of “normalcy” due to olipudase alfa was a source of distress for one caregiver. They expressed feelings of guilt due to the improvement in their child’s condition compared to other caregivers in the past whose ASMD-affected children did not have access to olipudase alfa and therefore experienced adverse disease courses. Based on their experience, this caregiver reported the view that all patients with ASMD need access to olipudase alfa.

“I mean there’s not “not” a need [for the therapy], right? You have a clear example in my child, in other children, and I hate saying it but the children that have passed. The pre-teens that have passed, the people that have passed. And that’s probably, without getting emotional, that’s probably one of the hardest parts of this journey for the last five years, four years, is seeing that my child is living a typical life. Our lives have returned to what you would call normalcy living because of this treatment for my daughter. And yet there have been other people that haven’t had that ability to access it that may have lost their lives because of it.” *Interview, 4 years and 11 months on treatment*

“We see that [name] is doing really well, there is not one symptom that we could wish to be further decreased. She has lots of energy, her belly is normal, she eats normal, she can play and do physical exercise like a normal 7-year-old girl. Also the doctors tell us that they could not have wished for any better result since all the parameters that are measured/followed up have gone towards normal a lot.”

*Survey, 3 years and 6 months on treatment*

“I have seen almost complete resolution of disease manifestations with the treatment.”

*Survey, 6 years and 1 month on treatment*

“Our daughter leads a life just like any other 7-year-old without ASMD and that’s all you ever want for your child.”

*Survey, 4 years and 11 months on treatment*

“Everything changed for [name], physically, mentally, it changed our family for the better.”

*Survey, 4 years and 11 months on treatment*

The caregiver with two children with ASMD included in this study also felt that access to olipudase alfa should be extended to all patients with the condition. They explained that only the younger of the siblings was initially accepted in the trial, and they went through the distress of knowing only one of their children would be receiving the treatment.

“We’re unique in which we had two kids going through the trial and our younger one was experiencing less symptoms because he got on early.”

*Interview, siblings started treatment at 7 years and 2 years of age, 4 years and 1 month and 6 years and 1 month on treatment, respectively*

This parent/caregiver outlined the unique challenge of having to explain to their five-year-old child who was not going to receive olipudase alfa that their younger sibling would continue to receive therapy that they perceived as beneficial. They also went through the distress of observing the child who received treatment at an early age improve with treatment while the other child’s physical, social, and emotional symptoms progressed. Additionally, the caregiver experienced conflicting emotions of happiness in seeing a child get better with treatment with the despondence associated with other sibling’s condition declining because they had no access to olipudase alfa. The caregiver remained hopeful that the positive results of the trial would mean their eldest child would eventually receive treatment as well. Of note, the sibling was eventually included in the trial.

“To pinpoint emotionally what we were going through was virtually impossible, because we had both ends of the spectrum. We had one kid who was able to do it and get the treatment so young where it had so much less of an impact on his body. And we were so hopeful in that sense. And yet we felt on the other end another kid knowing, which we saw physically what it was doing to him and emotionally and socially how he was struggling in school and so on. We were seeing all ends of every emotion possible."

*Interview, siblings started treatment at 7 years and 2 years of age, 4 years and 1 month and 6 years and 1 month on treatment, respectively*

When caregivers were asked to consider the prospect of losing access or stopping olipudase alfa, caregivers reported this scenario as dreadful and unthinkable. Caregivers suggested that the cessation of treatment could result in a complete reversal of the improvements seen in their children’s condition and QoL.

“It would be horrific. I cannot imagine not having it. It would... I don’t even know. I'm lost for words to say. If she didn't have access to it, it would be completely devastating. It, literally, has changed her life. She has been completely altered into a normal person living a normal life, all due to the treatment.”

*Interview, 5 years and 9 months on treatment*

“It’s something I don’t even want to think about. […] Without it, all those amazing things would go away. [Name] wouldn’t be [name] anymore she would be back to this fragile, sick, inability to walk, inability to run, inability to play, inability to go to school. All of those things that she can do now they would go away.”

*Interview, 4 years and 11 months on treatment*

## Discussion

This study sought to explore how a treatment that met predefined clinical endpoints in clinical trials impacted the burden of disease from the patient and caregiver perspective. Additional aims of the study were to better understand the perceived risk associated with olipudase alfa therapy and residual unmet therapeutic need by exploring the accounts of patients and families. Patients and families who have an extended period of exposure to a therapy are in a unique position to elaborate on the risk/benefit profile.

### Summary of findings

Both the qualitative and quantitative data in this study suggest olipudase alfa had a meaningful impact on the physical, emotional, and mental health of families.

The first theme that was generated in our study was the profound impact that olipudase alfa had on the physical symptoms of the disease. Importantly, olipudase alfa impacted disease-related morbidity in all areas and systems excluding the central nervous system that were referenced by participants. The qualitative component of this study demonstrates the impact of these symptoms on the broader QoL of patients, caregivers and their families.

The second theme to emerge was the impact olipudase alfa had on the emotional and mental health of caregivers. The stress associated with the uncertainty of prognosis, disease course, managing progressive care demands, and what the future will entail are among the many known stressors for families living with ASMD. These stressors extend beyond individual health to education, work, play and economic impacts.

Theme 3 provides significant insight about the risk associated with olipudase alfa from the patient and family perspective. This risk was considered minimal and associated with the inconveniences of participating in a clinical trial. The participants in this study clearly conveyed the benefits of the treatment outweighed any risk.

Participants in this study noted a meaningful treatment effect associated with this ERT on the non-neurologic manifestations of the disease but reported a continued unmet need regarding a treatment for the neurologic manifestations of the disease as detailed in Theme 4 above. Future research should be targeted at treating the Central Nervous System (CNS) manifestations of ASMD.

Theme 5 highlighted extensive unmet need across the ASMD community for a disease-modifying therapy. Participants in this study noted an urgent need for the entire community to have access to olipudase alfa based on their positive experience over a prolonged period of time. Olipudase alfa was just recently approved in only a handful of countries. At the time of this publication, access to this disease-modifying therapy is limited around the globe. Regulators, payors and the patient community need to collaborate to ensure widespread access to olipudase alfa for all ASMD patients based on the prolonged experience these patients and families have with this therapy.

### Comparison to previous literature

This patient-reported study clearly reaffirms an extensive and multi-faceted ASMD disease burden [7, 16] and provides a patient perspective on the mitigation of this burden through utilization of olipudase alfa.

The clinical studies evaluating both pediatric and adult patients on olipudase alfa achieved their primary outcomes in reduction in both spleen and liver size while also improving DLCO [11, 12]. Based on previously conducted studies, we are beginning to understand burden of ASMD for both patients and families including the known morbidity of abdominal/GI symptoms, fatigue, bleeding/bruising, physical function, social function and relationships to name a few [7, 16–18]. Morbidities experienced by patients in this study were reported across multiple body systems with gastrointestinal, skeletal and respiratory symptoms being among the most common sources of morbidity.

A previously conducted systematic review identified the significant psychological impact LSDs like ASMD have on families [18]. In that study, factors associated with the disease including disease severity and symptoms had a negative impact on parental outcomes. Uncertainty, poor prognosis, and clinical deterioration contributed to the disease’s negative psychosocial impacts. This report assimilates with our findings from Theme 2 which illustrates the extensive impact that ASMD has on families’ emotional, physical, and social health. Subsequently, the psychosocial wellbeing of families was improved in response to their child’s treatment with olipudase alfa.

Treatment of central nervous system manifestations is an unmet need across multiple LSDs including ASMD [19]. While caregivers in our study reported improvement in their child’s overall condition, most participants wished for a treatment which can permeate the blood brain barrier.

The psychosocial impact of inadequate access to treatment for all patients with ASMD has also been reported in a previously conducted study [20]. This unmet need is a source of frustration and anxiety in both patients and caregivers due to the knowledge a treatment is only currently available to a fraction of the ASMD patient population, leaving other patients vulnerable to adverse disease outcomes and unnecessary morbidity.

### Clinical and research implications

In rare disease trials, where the number of participants in a trial can be exceedingly small, this type of study allows for a richer understanding of the disease and the impact of an intervention. Capturing patient reported outcomes data retrospectively allows the trial participants to reflect over their entire life experience with the disease and provide a more comprehensive, informed narrative about how the intervention impacted the disease and its trajectory. This valuable information will be useful to patients, clinicians, researchers, industry, and payors.

By obtaining both qualitative and quantitative information, we were able to gain specific detail on previously understood morbidities of the disease but also gain detailed insights from each participant on other disease-associated symptoms that they noted from their own personal experience with the disease. Capturing the lived experience of each participant greatly enhanced the understanding of the morbidity of the disease and the changes each patient and family perceived while taking olipudase alfa.

The Niemann–Pick community, like other disease communities, has great interest in capturing patient-reported data to aid in research and clinical development. This type of retrospective study allows for the patient voice to be included as part of the outcome in a tangible way. This study highlights the physical and psychological impact ASMD has on QoL for patients and families. This information will be beneficial to the international ASMD community in many ways including their effort to capture the patient voice and patient experience in a longitudinal fashion through their international registry. In addition to enriching the understanding of the impact and effect of olipudase alfa on patients, this study will help guide future research on ASMD to address unmet clinical and supportive care needs. Continued patient reported research will be important to understand how these needs change as the natural history of ASMD evolves with treatment. The member organizations who supported this study are collaborating to replicate a similar study in the adult ASMD population to better understand the adult disease experience and impact of ERT. In summary, this study highlighted outcomes that are important to ASMD patients and families when considering therapeutic options, and this information will be valuable to guide further therapeutic development.

### Strengths and limitations

Strengths of the study include the wide geographic distribution of patients, the prolonged exposure to olipudase alfa, and the high percentage of patients included in this PRO study relative to the number of patients enrolled in the original olipudase alfa clinical trial. The consistency of the results is encouraging from the perspective of generalizing the study findings to the wider ASMD pediatric community. Additionally, the use of both quantitative and qualitative data in this study allows for a holistic examination of the patient and family perspective regarding olipudase alfa treatment.

Limitations include the retrospective nature of the study. Symptoms were not assessed prior to initiating olipudase alfa, so recall bias and the possibility of confounding are both possible. Given the lack of any other disease modifying therapy that is either approved or in the therapeutic pipeline, the risk of confounding is thought to be low. Pediatric patients were not interviewed in this study to avoid any potential for trauma through participation in the study. While the study sample size was small, it was limited to the small target population with only 20 patients in the pediatric clinical trial [11]. To mitigate real or perceived risk of bias in data acquisition and analytics, RDRP, an independent third-party research organization was contracted to complete interviews and analyze the responses. The study was funded by multiple advocacy organizations and unrestricted grants. There was no industry involvement in developing the study design, conducting the study, interpreting the results, or writing the manuscript.

## Conclusion

This study explored the lived experience of pediatric patients and families who have a prolonged experience with olipudase alfa as a potential therapy for ASMD. Olipudase alfa is being approved in some countries as the first disease modifying therapy for patients with this progressive LSD. This work contributes to the understanding of the unmet needs in this patient community, and it appears olipudase alfa successfully improved the physical, social and mental morbidity in patients and caregivers over a prolonged period of time with the exception of the neurologic symptoms of the disease. It was interesting that even though the neurologic symptoms were not perceived to be impacted by this disease, it was still effective in improving the social, mental and physical health of all patients and families by addressing the non-neurologic symptoms of the disease.

## Data Availability

The datasets generated and/or analyzed during the current study are not publicly available due to maintaining participant confidentiality and anonymity but are available from the corresponding author on reasonable request.

## Abbreviations

ASM: Acid sphingomyelinase
ASMD: Acid sphingomyelinase deficiency
PRO: Patient reported outcome
DLCO: Diffusion capacity for carbon monoxide
LSD: Lysosomal storage disorders
QoL: Quality of life
ERT: Enzyme replacement therapy
CNS: Central nervous system
